# Screening of Breast among Women: A Cross-Sectional Study in Nepal

**DOI:** 10.1155/2024/9969169

**Published:** 2024-05-20

**Authors:** Sangam Shah, Krishna Dahal, Paras Pangeni, Sandhya Niroula, Kiran Paudel, Prativa Subedi, Sarita Dhakal, Prince Mandal, Laba Rawal, Nikita Bhatta, Anisha Shrestha, Ganesh Bhattarai, Pragya Bhandari

**Affiliations:** ^1^Tribhuvan University, Institute of Medicine, Maharajgunj 44600, Nepal; ^2^Central Department of Public Health, Maharajgunj 44600, Nepal; ^3^Nepal Health Frontiers, Kathmandu, Nepal; ^4^Department of Allied Health Sciences, University of Connecticut, Storrs, CT, USA; ^5^KIST Medical College and Teaching Hospital, Imadole, Lalitpur, Nepal; ^6^Birat Medical College and Teaching Hospital, Biratnagar, Nepal

## Abstract

**Background:**

Breast cancer ranks as the second most prevalent malignancy among women in Nepal. This cancer has a high likelihood of cure, if detected early. Therefore, it is imperative to emphasize awareness and screening for breast cancer in Nepal. It indeed underscores the importance of clinical breast examination. The study aims to find the disease burden, association of abnormal breast condition with sociodemographic variables, and the need for change in the breast cancer screening protocol.

**Methods:**

A cross-sectional study was conducted from July to September 2023 on 100 female participants who were older than 18 years. Data were collected through face-to-face interviews using a structured questionnaire. The chi-square test was used to compare nominal variables while the independent sample *t*-test and the paired sample *t*-test were used to compare nominal and continuous variables.

**Results:**

The findings of the study showed that 19% of all participants complained about abnormal breast, out of which 31.7% reported lumps, 26.31% reported discharge , another 26.31% reported pain, and the remaining 15.7% reported soreness. The upper outer quadrant and lower inner quadrant each individually accounted for 33.33% of the abnormal findings. The complaints of the participants in our study were significantly correlated with age at marriage, number of pregnancies, and use of contraception.

**Conclusion:**

Our study revealed considerable abnormal breast findings. This warrants the need for the change in breast cancer screening protocols which lead to early diagnosis and higher curability.

## 1. Introduction

Breast cancer is a significant global health issue that has been steadily increasing over the past decade. Breast cancer was the second most common cancer among women in Nepal in 2012, after cervix cancer, according to data from seven major cancer service hospitals [1, 2]. In the year 2012, GLOBOCAN estimates that there were 1,700 new cases of breast cancer diagnosed in Nepal, with an age standardized rate (ASR) of 13.7 new cases per 100,000 women, and 870 female fatalities, with an ASR of 7.2 fatalities per 100,000 women [3]. The WHO has recommended that early detection of breast cancer can improve breast cancer outcome and survival [4]. The five-year survival rate is 90% in the first stage, 75% in the second stage, 50% in the third stage, and <10% in the fourth stage [5]. For the early detection of breast cancer, regular screening of all women is necessary. There are three methods of screening, i.e., breast self-examination, clinical breast examination, and mammography, and these are usually done in combination [6].

There is a huge challenge for nations with limited resources, where there are few facilities for therapy and diagnostics and where the cost of appropriate care for breast cancer typically exceeds monthly income [7]. The majority of breast cancer patients receive their diagnosis at a late stage, which makes treatment more challenging and less successful, according to several studies conducted in environments with limited resources [8, 9]. Individual factors (low breast cancer awareness and knowledge, myths and misconceptions, mistrust in the health care system, and financial and access barriers) and systemic factors (poor quality and availability of health care services, absence of specialized public services and necessary drugs, high diagnosis and treatment costs, and low breast cancer awareness) have been identified as contributing factors to late presentation to health care providers [9–13]. In high-income nations, breast cancer screening programs are widely available; however, in low- and middle-income countries (LMICs), where treatment options are limited locally and the population is young, there are few or no screening programs and they are likely to be both ineffective and expensive [14]. The current state of breast cancer screening in Nepal is characterized by lack of resources and awareness, particularly in rural areas [15].

To guide the development, expansion, and strengthening of health care system and breast cancer screening programs, it is crucial to estimate the prevalence of breast symptoms which need diagnostic service and likely follow-up and treatment [16]. However, there are very few literatures which cover the findings of the breast screening in our country. Therefore, the current study aimed to assess the prevalence, clinical profile of breast diseases, and their association with sociodemographic variables among females in the Tokha Municipality of Nepal. The findings of the study are very essential in regards to see the disease burden, association of abnormal breast condition with sociodemographic variables, and the need for change in the breast cancer screening protocol.

## 2. Methodology

### 2.1. Design and Setting

A cross-sectional study was conducted in Tokha Municipality on 100 female participants. Among 11 wards in Tokha, 4 wards were chosen randomly. The random sampling method was employed in the chosen wards. Randomly selection was carried out to minimize sampling bias and to ensure representation of the population.

### 2.2. Sample Size

If,

*n* = sample size.

*z* = level of confidence measure, if *α* = 0.05, *Z* = 1.96 for 95% confidence interval.

*p* = baseline prevalence incidence of major complication (*p*) = 5.8%.

*d* = margin of error. For this study, we have taken *d* = 5%, *d* = 0.05.

Then, simple size (*n*) can be calculated as *n* = *z*^2^*p* (1 − *p*)/*d*^2^ = [(1.96)^2^ *∗* 0.056 *∗* 0.944]/(0.05)^2^ = 81.

So, the sample size is about 81 participants.

We included around 100 participants.

### 2.3. Study Subjects and Participants

Female aged over 18 years and who were willing to participate in the study were included in the study. Female younger than 18 years and those who did not give informed consent were excluded.

### 2.4. Outcome Measurement and Variables

The dependent variables were characteristic of complaints and behavioral characteristics, while the independent variables were sociodemographic characteristics.

### 2.5. Ethical Consideration

Ethical approval was obtained from the Research Ethics Committee of the Nepal Health Research Council (approval number: 2915). Written informed consent was obtained from the study participants for the use of anonymous personal and clinical data in research after full explanation about the purpose and outcome of research. Confidentiality of the information was maintained thoroughly by deidentification.

### 2.6. Data Collection and Study Variables

Data were collected through face-to-face interviews using a structured questionnaire from July to September 2023. Validity of the questionnaire was ensured by the reviewers having the expertise in this field. Reliability analysis was conducted on the questionnaire responses. The questionnaire underwent pretesting in 10% of the sample size.

Data collection was done by the principal researcher along with three trained research assistants with a public health background. Medical doctors from Maharajgunj Medical College also assisted in data collection in Tokha Municipality. All the enumerator went through comprehensive orientation session where the study protocol, methodologies, and the technique of self-breast examination to the participants were thoroughly explained. The enumerators taught the respondent the correct technique to identify abnormalities. The respondents were then asked to perform the same technique on their own and if they reported any abnormalities they were sent for radiological examinations. In addition to suggesting clinical and cytological diagnoses for reported breast abnormalities, this study offered 100 women the chance to learn about breast abnormalities. A normal breast was painless, had clear skin, no lumps, a normal temperature, no discharge, and normal or abnormal mobility. Abnormal breasts were any divergence from the normal characters.

The variables selected include a wide variety of sociodemographic and behavioral variables that are known to affect breast health. These variables were chosen to capture different aspects, including age-related susceptibility, cultural biases, lifestyle habits, reproductive background, and health-seeking behaviors. The goal of this study is to understand the intricate interactions between these variables and breast abnormalities.

### 2.7. Statistical Analysis

Data were entered in Microsoft excel version 2019 and data analysis was performed on IBM Statistical Package for the Social Sciences (SPSS) version 21. Descriptive analysis was done by calculating frequency and percentages of categorical variables. The chi-square test was employed for the comparison of nominal variables, while the independent sample *t*-test and paired sample *t*-test were utilized for the comparison of nominal and continuous variables.

## 3. Result

### 3.1. Sociodemographic Characteristics of Study Population

Table 1 displays the sociodemographic details of research participants. Of the total 100 participants, 51% of them were older than 40, while the remaining were younger. The majority of participants in the study were married. A considerable percentage (54%) was illiterate.

### 3.2. Behavioral Characteristics of Study Population

The findings reveal that 28% of the individuals smoke and 24% drink alcohol on a regular basis. Of the participants, 35% began their menstrual cycle before the age of 13, 62.5% got married before turning 20, and 87.8% had their first sex before the age of 20. Of them, 53% were still sexually active (Table 2).

### 3.3. Characteristics of the Complaints

Of the total participants, 19% complained about the abnormal breast. Out of which 31.7% reported lumps, 26.31% reported discharge, another 26.31% reported pain, and the remaining 15.7% reported soreness as shown in Table 3.

Relationship between the complaints and sociodemographic and characteristics of the respondents.

There is a significant correlation between the age at first marriage and the complaints, specifically 18.8% of all participants who married before the age of 20 had abnormal cases, with a *P* value of 0.04, as shown in Table 4.

### 3.4. Factors Associated with the Complaints

There was a significant relation between use of contraception and abnormal breast (*P* value 0.02), as shown in Table 5.

## 4. Discussion

Our finding revealed that 19% of the total participants complained about abnormal breast. Out of which 31.7% reported lumps, 26.31% reported discharge , another 26.31% reported pain, and the remaining 15.7% reported soreness. The upper outer quadrant and lower inner quadrant each individually accounted for 33.33% of the abnormal findings. In our study, variables such as age at marriage, number of pregnancy, and contraception showed significant association with the complaints of the participants. In this study, we have projected the need for the change of breast cancer screening in our health system since a substantial proportion of the participants reported abnormal breast findings.

In our study, 19% of the participants reported abnormal breast which is similar to the study of Malmartel et al., where 22.2% had an abnormal findings [17]. Ayele et al. showed less participants complaining of abnormal breast [16, 19, 20]. This might be because in the study by Ayele et al., clinical examination was performed only on the symptomatic patients which might have shown lower disease burden. In our study, lump and discharge accounted for major complains unlike pain and lump which was common in the studies by Ayele et al., Ayoade et al., Alamri et al., and Marmartel et al. [17, 19, 21, 22]. These suggest varying presentations of breast abnormalities. In the study by Celene et al., most common presentation of breast cancer was painless lump while other presentation were pain, inverted nipple, boil, and abnormal breast size [23].

In the same way, in our study, use of contraception was significantly associated with abnormal breast findings, which was consistent with the study in Ethiopia by Ayele et al. [19]. In a meta-analysis of several studies has shown a slight increased risk of abnormal breast findings ranging from 8 to 24% [24, 25]. Early studies found no association between contraceptives and breast cancer, with Kelsey even suggesting a potential protective effect [18, 26]. However, more recent research has documented an increased risk of breast cancer among hormonal contraceptive users although the overall risk remains low. These findings highlight the need for further research to fully understand the impact of contraception on breast health.

In the study of Ayele et al., age at first menstruation was significantly associated with abnormal breast findings whereas in our study, it was not significant [19]. This study's findings on abnormal breast findings were significantly correlated with the use of contraception, age at first marriage, and number of pregnancies similar to the study of Bk et al. [27]. Early marriage, particularly before the age of 18, has been identified as a potential risk factor for breast cancer in the Eastern Region of Saudi Arabia [4]. This is consistent with the broader association between marital status and cancer diagnosis, with unmarried individuals being more likely to be diagnosed at a later stage [28]. Similarly, parity, or the number of pregnancies, is linked to a short-term increase in breast cancer risk, followed by a long-term decrease [29]. These findings suggest that hormonal risk factors may be contributing. This suggests that more research is necessary to determine how these are related to one another.

In addition to suggesting clinical and cytological diagnoses for reported breast abnormalities, this study offered 100 women the chance to learn about breast abnormalities. The findings of the study have shown the burden of breast abnormalities and unfulfilled diagnostic needs across the country. This warrants the need of change in breast screening protocols as routine mammography screening in a resource‐limited country with a young population is neither sensitive nor affordable. The data of the study will serve as a policy guide to improve breast diagnostic and treatment.

Our study also had some limitations. The data were collected by self-report which can introduce biases as participants may not accurately recall or report their experiences, leading to underreport or overreport of the symptoms. This can result in inaccurate prevalence estimates. Since the sample size is relatively small, so the result cannot be generalized as it might not accurately represent the true prevalence or association; hence, it is recommended to conduct further studies using larger samples at various areas. We were not able to incorporate all the factors that could have influenced the association between the variables. Despite the limitations, the findings of this study highlighted that there is a critical need for reinforcing breast cancer screening protocols and resource allocation in health system.

## 5. Conclusion

Our study has identified a high prevalence of breast abnormalities that require immediate action. It is very important to prioritize education and awareness programs aimed at promoting early detection of breast abnormalities. Policymakers must tailor screening protocols to address the unique needs of different demographic groups and ensure the timely availability of diagnostic services, including clinical and radiological examinations, particularly in underserved areas.

The study's findings offer constructive insights for healthcare practitioners in Nepal. By utilizing these insights, healthcare providers can tailor their approaches to identify and manage breast abnormalities among women more effectively. Through prioritizing awareness campaigns, early detection efforts, and improved access to diagnostic services, healthcare providers can significantly enhance breast health outcomes and reduce the burden of breast abnormalities in the population. Given the limitations of routine mammography screening in resource-limited settings with a predominantly young population, policymakers should explore alternative screening modalities that are more sensitive, cost-effective, and feasible in the Nepalese context. This may include the integration of breast self-examination and clinical breast examination into primary healthcare services. It is absolutely necessary to invest in healthcare infrastructure, training of healthcare personnel, and strengthening referral systems to ensure timely follow-up and treatment for women with abnormal breast findings.

## Figures and Tables

**Table 1:** Sociodemographic characteristics of the study participants (*n* = 100).

	Number (percentage)
*Age in years*	
≤40 years	49 (49)
>40 years	51 (51)

*Religion*	
Hindu	62 (62)
Others	38 (38)

*Occupation*	
Housewife	38 (38)
Farmer	46 (46)
Others	16 (16)

*Ethnicity*	
Brahmin	17 (17)
Chhetri	19 (19)
Janajati	57 (57)
Others	7 (7)

*Education*	
Illiterate	54 (54)
Primary	23 (23)
Midschool	11 (11)
High school and above	12 (12)

*Married*	
Married	96 (96)
Unmarried	4 (4)
Widow	7 (7)

**Table 2:** Behavioral characteristics of the study participants (*n* = 100).

	Numbers (percentage)
*Smoking*	
Yes	28 (28)
No	72 (72)

*Alcohol*	
Yes	24 (24)
No	76 (76)

*Age at first menstruation*	
≤13	35 (35)
>13	65 (65)

*Age at first marriage*	
≤20	60 (62.5)
>20	36 (37.5)

*Sexually active*	
Yes	53 (53)
No	47 (47)

*Age at first sex*	
≤20	86 (87.8)
>20	12 (12.2)

*Number of pregnancy*	
≤2	37 (37.8)
>2	61 (62.2)

*Number of children*	
≤2	42 (42.9)
>2	56 (57.1)

*Comorbid*	
Yes	30 (30)
No	70 (70)

4 of the respondents were unmarried. 2 of the respondents didn't have sex. 2 of the respondents didn't have any pregnancy. 2 of the respondents didn't have any children.

**Table 3:** Characteristics of the complaints.

	Numbers
Complaints	
Normal	81 (81)
Abnormal	19 (19)
Abnormality	
Lump	6 (31.57)
Discharge	5 (26.31)
Pain	5 (26.31)
Soreness	3 (15.7)
Lump	
Size	
0.5 *∗* 0.5	1 (16.66)
1 *∗* 2	1 (16.66)
2 *∗* 1	1 (16.66)
2 *∗* 1.5	1 (16.66)
2 *∗* 3	1 (16.66)
3 *∗* 2	1 (16.66)
Shape	
Irregular	2 (33.33)
Oval	4 (66.7)
Number	
1	100 (100)
Mobility	
Mobile	4 (66.7)
Nonmobile	2 (33.3)
Quadrant	
Upper outer	2 (33.33)
Upper inner	1 (16.66)
Lower outer	1 (16.66)
Lower inner	2 (33.33)
Side	
Right	7 (46.7)
Left	8 (53.3)
Colour of discharge	
White	2 (40)
Red	2 (40)
Yellow	1 (20)
Types of discharge	
Mucoid	3 (60)
Serous	2 (40)
Tenderness	
Tender	5 (31.25)
Nontender	11 (68.75)
Side Bilateral	1

**Table 4:** Relationship between the complaints and sociodemographic and characteristics of the respondents.

Variable	Normal	Abnormal	*P* value
*Age in years*			
≤40 years	41 (83.7)	8 (16.3)	0.9
>40 years	43 (84.3)	8 (15.7)	

*Religion*			
Hindu	49 (79)	13 (21.)	0.08
Others	35 (92.1)	3 (7.9)	

*Occupation*			
Housewife	31 (81.6)	7 (18.4)	0.7
Farmer	40 (87.0)	6 (13.6)	
Others	13 (81.3)	3 (18.8)	

*Ethnicity*			
Brahmin	12 (70.6)	5 (29.4)	0.4
Chhetri	16 (84.2)	3 (15.8)	
Janajati	50 (87.7)	7 (12.3)	
Others	6 (85.7)	1 (14.3)	

*Education*			
Illiterate	46 (85.2)	8 (14.8)	0.2
Primary	21 (91.3)	2 (8.7)	
Midschool	9 (81.1)	2 (18.2)	
High school	2 (50)	2 (50)	
+2 and above	8 (66.7)	4 (33.3)	

*Marital status*			
Married	76 (85.4)	13 (14.6)	0.3
Unmarried	3 (60.0)	2 (40.0)	
Widow	5 (83.3)	1 (16.7)	

*Smoking*			
Yes	25 (89.3)	3 (10.7)	0.3
No	59 (81.9)	13 (18.1)	

*Alcohol*			
Yes	20 (83.3)	4 (16.7)	0.9
No	64 (84.2)	12 (15.8)	

*Age at first menstruation*			
≤13	29 (82.9)	6 (17.1)	0.8
>13	55 (84.6)	10 (15.4)	

*Age at first marriage*			
≤20	65 (81.3)	15(18.8)	**0.048**
>20	16 (100)	0 (0)	

*Sexually active*			
Yes	44 (83.0)	9 (17)	0.7
No	40 (85.1)	7 (14.9)	

*Age at first sex*			
≤20	70 (81.4)	16 (18.6)	1.02
>20	12 (100)	0 (0)	

*Number of pregnancy*			
≤2	34 (91.9)	3 (8.1)	**0.045**
>2	48 (78.7)	13 (21.3)	

*Number of children*			
≤2	36 (85.7)	6 (14.3)	0.6
>2	46 (82.1)	10 (17.9)	

*Comorbid*			
Yes	23 (79.3)	6 (20.7)	0.4
No	61 (85.9)	10 (14.1)	

*Menstruation*			
Regular	26 (81.3)	6 (18.8)	0.6
Other	58 (85.3)	10 (14.7)	

*Contraception*			
Yes	27 (75)	9 (18)	**0.049**
No	56 (88.9)	7 (11.1)	

The bold values are statistically significant data.

**Table 5:** Factors associated with the complaints.

Variable	Unadjusted odds ratio (95% CI)	*P* value	Adjusted odds ratio	*P* value
*Religion*				
Hindu	3.8 (0.9–15.6)	0.06	3.0 (0.8–11.6)	0.09
Others	Ref		Ref	

*Contraception*				
Yes	0.2(0.08–0.8)	**0.02**	0.3 (0.1–1.1)	0.07
No	Ref			

*No of pregnancy*				
≤2	3.1 (0.7–12.3)	0.1	3.06 (0.8–11.6)	0.098
>2	Ref			

The bold value signifies statistically significant values.

## Data Availability

The data used to support the findings of this study are included within the article.
